# Generational compression and the shifting anchors of identity: a photo-elicitation nostalgia narrative study of identity construction across three generations in an Oroqen family

**DOI:** 10.3389/fpsyg.2026.1915280

**Published:** 2026-09-25

**Authors:** Shan Cong

**Affiliations:** Department of Chinese Language and Literature, Sun Yat-sen University, Guangzhou, China

**Keywords:** generational transfer, identity anchors, language endangerment, mutual constitution, nostalgia narrative, Oroqen, photo-elicitation interviewing

## Abstract

Drawing on the mutual constitution framework in cultural psychology, this study explores how three generations of an Oroqen family construct ethnic identity through nostalgic narratives elicited by family photographs. The Oroqen, one of China’s smallest ethnic minority groups, with a population of only 9,168, offer a rare example of rapid cultural change. In the span of only three generations, or roughly 80 years, they moved from a forest-based hunting and gathering way of life into full participation in a digital society. Using reflexive thematic analysis, the analysis examined in-depth interviews with five members of the family and identified three core themes. First, the main anchors of ethnic identity shifted across generations. For the grandparents, Oroqen identity was rooted in embodied memories of hunting and gathering. The parent generation centered it on preserving traditions that were fading away. The grandchild generation, by contrast, reconstructed identity through selective symbols and digital communication. Second, the link between language ability and ethnic identity changed across generations, moving from essentialist claims to pragmatic compromise and then to more fluid forms of reconstruction. Each generation also bore its own emotional burden in this process. Third, digital media created new possibilities for the grandchild generation to reshape ethnic identity. This gave rise to an intergenerational dynamic in which a decline in lived experience was accompanied by a widening range of communication. These findings extend the mutual constitution framework by showing how generational position and media environments shape identity construction. The study also highlights the value of photo-elicitation interviews for capturing generational differences in identity construction in culturally endangered contexts.

## 1 Introduction

The Oroqen, one of China’s smallest ethnic minority groups, are an Indigenous people of the Northern Tungusic branch. Historically, they inhabited the Heilongjiang (Amur) River basin and the Greater and Lesser Khingan Mountains, relying on hunting and gathering for subsistence. Following the founding of the People’s Republic of China, the Oroqen were officially recognized as one of the country’s 56 ethnic groups and today have a population of only 9,168 (National Bureau of Statistics of China, 2022). Over roughly 80 years, or just three generations, the Oroqen have undergone an unusually compressed process of cultural change. The state-led sedentarization policy introduced in 1953 ended their nomadic hunting life. The comprehensive hunting ban in 1996 brought that tradition to a close. Since the turn of the century, the spread of digital media has further reshaped their forms of cultural expression. UNESCO classifies the Oroqen language as severely endangered (Moseley, 2010). The Oroqen case therefore provides a particularly strong test of the mutual constitution principle. Here, cultural resources are not only changing but, in some cases, disappearing. As a result, successive generations must draw on very different forms of experience to answer the same question: what makes someone Oroqen?

In cultural psychology, the mutual constitution principle holds that culture and individual psychology are continuously shaped through their dynamic interplay. Culture provides resources for the self through language, institutions, and shared systems of meaning, while individuals sustain, reshape, and sometimes contest these resources in everyday life (Markus and Kitayama, 1991, 2010; Shweder, 1991). Recent scholarship has shown that this process is far more complex than earlier models suggested. People no longer form the self within a single, stable cultural system. Instead, they negotiate identity at the intersection of multiple cultural resources: local and global, online and offline, inherited and chosen (Hermans and Dimaggio, 2007; Jensen, 2003; Appadurai, 1996). Given the scarcity of documentary and textual records within Oroqen families, photographs and the memories they evoke offer a particularly valuable point of entry into how this negotiation of identity unfolds across generations.

This study therefore uses a photo-based nostalgic narrative approach (Elbaz and Wiseman, 2026), building on the tradition of photo-elicitation interviewing (PEI) (Harper, 2002; Clark-Ibáñez, 2004; Pink, 2013), as photographs can prompt recollection of embodied practices and emotionally significant events that verbal interviewing alone may not readily access. It examines how five members of a three-generation Oroqen family in the Oroqen Autonomous Banner of Inner Mongolia anchor their ethnic identity through photo selection and narrative reflection. The study addresses three research questions:

RQ1: What identity anchors do different generations draw on in their photo selections and narratives to affirm their Oroqen identity?

RQ2: How are these identity anchors passed across generations in response to sedentarization, the hunting ban, language loss, heritagization, and digital media?

RQ3: How do the identity constructions of the three generations, despite their differences, work together to form a contemporary family-based ecology of ethnic identity?

This study is an exploratory case study of a single family. It does not aim to produce generalizable conclusions about the Oroqen as a whole. Rather, it seeks to explore a possible pattern in the intergenerational transmission of identity anchors, to show the value of photo-elicitation interviews for capturing generational differences in identity construction in culturally endangered contexts, and to consider how generational and media dimensions might extend the mutual constitution framework in cultural psychology.

## 2 Theoretical framework

### 2.1 Mutual constitution and identity co-construction in cultural psychology

The mutual constitution principle is a core idea in cultural psychology and was developed most systematically by Markus and Kitayama (1991, 2010) through their distinction between independent and interdependent self-construals. The independent self is characterized by autonomy, uniqueness, and personal achievement, whereas the interdependent self is defined by social relationships, group belonging, and relational harmony. This distinction provides the conceptual basis for understanding how Oroqen family members negotiate identity between traditional interdependent practices and the increasingly independent orientations associated with urbanization and mainstream schooling. Markus and Kitayama (2010) describe mutual constitution as a dynamic process in which cultural patterns shape how people construct the self, while everyday practices in turn reproduce and reshape those same cultural patterns. This principle has been widely supported in three decades of cross-cultural psychological research (Heine, 2001; Kitayama et al., 2009; Nisbett, 2003).

In identity research, the concept of identity co-construction (Bamberg, 2011; Schwartz et al., 2020) views identity as something continuously negotiated across social relationships, cultural contexts, and historical moments. Cultural psychologists have proposed several influential frameworks to explain this process. Benet-Martínez and Haritatos (2005) proposed the bicultural identity integration (BII) model, which distinguishes between cultural conflict and perceived cultural distance. Through research on cultural frame switching, Hong et al. (2000) showed that bicultural individuals display considerable cognitive flexibility when exposed to different cultural cues. Morris et al. (2015) advanced a polycultural psychology approach, arguing that cultures are inherently dynamic and constantly shaped through mixing and borrowing. Lu et al. (2023) further proposed a socioecological-genetic framework, suggesting that socioecology, including geographical environments and modes of subsistence, provides a shared foundation for both culture and personality. Foundational work by Berry (1997, 2005) established that acculturation involves changes at both group and individual levels, with four strategies: integration, assimilation, separation, and marginalization, shaping how individuals negotiate multiple cultural affiliations. Building on this foundation, Schwartz et al. (2020) extend Berry’s model by emphasizing the dynamic, bidirectional, and identity-related dimensions of acculturation, which this study draws upon to interpret how successive generations within the Oroqen family reposition themselves relative to both Oroqen and Han Chinese cultural practices.

At the level of identity motives, Vignoles et al. (2016) identified six universal motives in large-scale cross-cultural survey research, while Becker et al. (2018) showed that motives for self-continuity vary across 55 cultural groups in culturally flexible ways. Drawing on a semiotic framework, Cousins (2012) highlighted intentionality as a key mechanism linking individual psychology to cultural symbol systems. Thomsen et al. (2007) used a relational practice framework to examine how social relationship structures shape cultural psychological differences. Mitchell et al. (2021) further showed that identity integration continues to develop throughout adulthood in response to role transitions and changing cultural environments. Using acculturation as a primary example, Schwartz et al. (2020) illustrated the convergence between cultural psychology and developmental science. They argued that identity construction occurs not only in contexts of intercultural contact but also through generational differentiation produced by historical change within a single culture. This insight speaks directly to the present study, which examines generational differences in identity under the unusually compressed cultural change experienced by the Oroqen.

### 2.2 Narrative identity, nostalgia, and self-continuity

Narrative identity is a central concept in contemporary personality psychology. In his life story model, McAdams (1985, 2001, 2008) proposed that identity is an internalized and evolving narrative that brings together the past, present, and future into a meaningful whole. He also emphasized that this narrative is shaped by culturally available story templates, such as redemption and contamination. Bruner (2004) extended this view by arguing that life itself has a narrative structure. People do not first live an objective life and then describe it in language. Rather, experience is organized, understood, and given meaning through narrative frameworks from the very beginning.

Habermas and Bluck (2000) introduced the concept of autobiographical reasoning and identified four forms of narrative coherence: temporal, causal-motivational, thematic, and cultural. This framework provides a useful way to analyze identity construction in narrative texts. Habermas and Köber (2015) showed that autobiographical reasoning can reduce the negative effect of biographical disruption on self-continuity. Camia and Zafar (2021) confirmed this protective function among refugee populations, but also identified an important limit: when life disruption exceeds a certain threshold, the effect can reverse.

Building on this understanding of autobiographical reasoning and narrative coherence, recent research has increasingly examined how emotionally charged recollections of the past are organized into meaningful experience, with nostalgia emerging as one such mechanism in social psychology. Nostalgia, defined as a sentimental longing for one’s personal past (Sedikides et al., 2015), has been shown to play a causal role in restoring self-continuity. Negative self-discontinuity can trigger nostalgia, and nostalgic recollection in turn strengthens self-continuity. This effect holds independently of positive affect. Hong et al. (2022) further identified the narrative pathway through which this process unfolds. Hepper et al. (2014) showed that nostalgia has cross-cultural relevance across 18 countries, while Pickering and Keightley (2006) highlighted its multiple dimensions, including both a melancholic tone and a utopian impulse. Beyond self-continuity, nostalgia also helps people access the inner self (Baldwin et al., 2015), strengthens meaning in life (van Tilburg et al., 2019), supports online identity construction (Dai et al., 2021), and functions as a broader psychological resource (Routledge et al., 2013). In terms of narrative structure, Steiner et al. (2017) found that the emotional tone of life story chapters anchors self-concept more stably than specific memories do. Enz and Talarico (2016) further highlighted the role of turning point memories in organizing life timelines and giving meaning to identity.

These findings provide the main theoretical foundation for the present study. In this study, the nostalgia narratives elicited by photographs from three generations of an Oroqen family are understood as efforts to restore identity continuity under conditions of severe cultural rupture. This process involves both the restoration of self-continuity at the individual level and the collective protection and intergenerational transmission of cultural memory.

### 2.3 Photo-elicitation interviewing as a visual method in cultural psychology

Photo-elicitation interviewing (PEI), first introduced by Collier (1957) and later developed by Harper (2002) and Pink (2013), is now a well-established method in qualitative research. The basic idea behind PEI is that photographs are not just interview aids. They also work as boundary objects that can evoke deeper layers of memory, emotion, and cultural awareness. Harper (2002) noted that photographs engage cognitive and emotional processes that differ from those prompted by verbal discussion. As a result, they often bring out information that interviewers cannot reach through words alone.

Photographs serve several purposes in identity research. Van Dijck (2008) identified three overlapping functions of personal photography: communication, identity, and memory. She showed that digital technology has shifted personal photography from a tool for preserving memory to one for constructing identity and social communication. Clark-Ibáñez (2004) distinguished between photographs provided by researchers and those taken by participants. The present study draws on both approaches. Pink (2013) located PEI within the broader tradition of visual ethnography. She argued that visual methods do not simply record culture but also participate in shaping it. From this perspective, the standardized photo set provided by the researcher is not a neutral measurement tool but a deliberately created space for cultural dialogue. From a constructivist perspective, Jiménez-Alonso and Brescó de Luna (2022) examined how photography helps generate meaning during life transitions. Brescó de Luna (2016) further argued that autobiographical memory is not a fixed retrieval of the past, but an ongoing narrative reconstruction shaped by social interaction and mediating tools.

Two later developments further extended this methodological tradition. Wang and Burris (1997), later discussed by Catalani and Minkler (2010), developed Photovoice as a participatory visual method. Elbaz and Wiseman (2026) proposed the photo-elicitation nostalgia narrative approach adopted in the present study.

PEI is especially valuable in cross-generational research. In Indigenous and endangered cultural contexts, photographs of traditional practices and ways of life can prompt participants to reflect on and express their cultural identity. For groups affected by language loss or displacement, photographs also provide a way of communication that goes beyond what verbal interviewing alone can achieve.

### 2.4 Chinese ethnic policy, language endangerment, and Oroqen studies

To understand Oroqen identity construction, it is necessary to place it within the institutional framework of Chinese ethnic policy. The ethnic classification project (*minzu shibie*) of the 1950s assigned clear institutional identities to groups. This process profoundly shaped minority self-understanding and their relationship with the state (Mullaney, 2011; Harrell, 1995; Gladney, 2004).

Grenoble and Whaley’s (1999) comparison of Chinese and Soviet Tungusic language policies revealed an important paradox. China’s relatively accommodative ethnic policies encouraged high rates of interethnic marriage and integration into mainstream society. At the same time, they also unintentionally accelerated the decline of intergenerational language transmission. Janhunen (2005) further noted that Tungusic languages in China face more serious endangerment than those in Russia. The changing ethnic backgrounds of spouses across the three generations in this study, from Oroqen to Han to Daur, offer a family-level example of this broader pattern.

The relationship between language loss and ethnic identity is a central issue in this literature. In their study of the Australian Indigenous language, Gurindji, Bromham et al. (2020) found that peer group was the strongest predictor of language variation, even more influential than the family environment. They also showed that monolingual mainstream education accelerated language loss, with productive ability declining faster than receptive ability. Through qualitative research with Indigenous Australians, Neville et al. (2014) identified five core dimensions of belonging: history, memory, and place; sense of community; acceptance and pride; shared language and culture; and interconnectedness. Importantly, these dimensions can compensate for one another. When shared language is weakened at a structural level, other dimensions may become stronger and help sustain an overall sense of belonging. This provides a useful cross-cultural reference for understanding how younger Oroqen may construct ethnic identity without fluency in their ancestral language. Using national survey data, Biddle and Swee (2012) found a significant positive association between connection to traditional land, participation in traditional subsistence activities, and subjective well-being.

Beyond these interpersonal and practice-based mechanisms of cultural transmission, language itself warrants specific attention, as it is frequently treated as a defining marker of ethnic belonging. However, such framing in endangered language communities is better understood not as a direct reflection of cultural reality, but as a strategic discursive practice (Schwartz, 2018; Debenport and Webster, 2019). Indigenous languages also provide a psychological foundation for collective resistance (Droogendyk and Wright, 2017). In language revitalization research, Grenoble and Whaley’s (2021) Language Vitality Network Model challenges the traditional division between endangered and safe languages. They argue that language vitality is better understood as a dynamic part of broader networks of community well-being. Hinton’s (2001) discussed the value of fragmentary transmission as a meaningful starting point. Ka’ai (2017) offered a sobering reminder that language loss may take only one generation, whereas revitalization requires at least three.

Psychological research on identity among Chinese ethnic minorities, especially at the level of family processes, remains limited. Existing scholarship on the Oroqen has focused mainly on historical anthropology (Qiu, 1956; Zhao, 1991), linguistic description (Hu, 1986, 2001; Cong, 2019), and the protection of intangible cultural heritage (Wu, 2006; Guan and Wang, 2010). Psychologically oriented work remains scarce. From a cognitive psychological perspective, Wang et al. (2024) showed how hunting culture shapes verbal fluency and cognitive flexibility. Ni and Guo (2023) proposed that Oroqen shamanic culture has gone through processes of embedding, disembedding, and re-embedding. The present study seeks to address this gap by examining intergenerational identity construction within the family.

### 2.5 Theoretical integration of the present study

This theoretical framework integrates three interrelated strands: mutual constitution as a cyclical process linking culture and self (Markus and Kitayama, 1991, 2010); narrative identity and nostalgia as mechanisms through which individuals construct coherence and meaning from personal and collective memory (McAdams, 2001; Sedikides et al., 2015); and family narrative transmission as the interpersonal process through which cultural meanings are passed across generations (Zilber et al., 2008). Together, these strands provide a multi-level lens for analyzing how ethnic identity is co-constructed within the Oroqen family across time.

First, it extends the mutual constitution framework. Existing research has mainly examined the mutual constitution of culture and the individual (Markus and Kitayama, 2010). This study adds a generational perspective and shows how rapid historical change can produce different identity anchors within the same culture.

Second, it applies narrative identity and nostalgia theory to the study of endangered cultures. It also extends PEI from a method centered on single participants to a tool for cross-generational comparison.

Third, it connects the family narratives of the Oroqen to broader institutional analysis (Grenoble and Whaley, 1999; Janhunen, 2005). In doing so, it shows how the policy paradox described above is lived, adapted to, and negotiated in the identity practices of one family.

Through this integration, the study uses identity anchors as a unifying concept. It brings generational and media dimensions into the mutual constitution framework and contributes empirical evidence from a Chinese indigenous context to the broader literature on identity construction in endangered language communities.

## 3 Methods

### 3.1 Research design

This study adopts a constructivist paradigm (Lincoln et al., 2011) and uses an in-depth single-family case design. It combines photo-elicitation interviewing (Harper, 2002; Clark-Ibáñez, 2004; Pink, 2013), reflexive thematic analysis (Braun and Clarke, 2006, 2019), and narrative analysis (Josselson and Hammack, 2021). The single-family case design was chosen because it allows close examination of how identity anchors are passed on and reshaped across generations. It was not intended to support statistical generalization. Focusing on the narratives of a single family makes it possible to trace the specific ways cultural resources are transmitted, contested, and renegotiated. This design also helps capture relational dynamics that cross-family comparative studies are less able to show.

### 3.2 Research setting, family background, and fieldwork foundation

The study was conducted in the Oroqen Autonomous Banner in Hulunbuir, Inner Mongolia Autonomous Region, China. Established in 1951 as China’s first ethnic autonomous banner, it remains one of the main areas of Oroqen settlement and has an Oroqen population of about 3,000. Using purposive sampling, the researcher selected a family whose members had lived in the banner across three generations. The family was chosen because it was both typical and information-rich. It was typical in that its members had lived through the major social changes of the past half-century: sedentarization in the grandparent generation, educational modernization and the hunting ban in the parent generation, and heritagization and digitalization in the grandchild generation. It was also information-rich because the three generations differed substantially in age, gender, education, occupation, and the ethnic backgrounds of spouses.

Since 2015, the sole author has conducted nearly ten years of documentation on Oroqen language and culture in the region, can communicate in Oroqen with native speakers, and has maintained a long-term relationship with the participating family. During the interviews, participants showed a high level of trust and shared feelings that were deep, ambivalent, and at times painful. The researcher’s familiarity with the family’s life histories, kinship networks, and community background also allowed for more context-sensitive interpretation of code-switching and emotional cues throughout the interviews.

### 3.3 Participants

Five members of the same family across three generations took part in the study. All names used here are pseudonyms.

Omon, a woman from the oldest generation, is the family matriarch and has two children: Ilan, her son, and Dijin, her daughter. Nadan is Ilan’s son, and Jejin is Dijin’s son. Omon’s spouse was Oroqen and is now deceased. The spouses of Ilan and Dijin are Han, Nadan’s spouse is Daur, and Jejin is unmarried. All three generations live in the Oroqen Autonomous Banner and meet frequently for festivals and important family occasions. Table 1 presents the participants’ demographic information.

**TABLE 1:** Participant demographic information.

Generation	Pseudonym	Gender	Age	family relation	Spouse’s ethnicity	Education	Occupation	Oroqen proficiency	Key life events	Media use
Grandparent	Omon	F	80	Family matriarch	Oroqen (deceased)	Limited formal education	Banner unit (retired)	Fluent native speaker	Experienced sedentarization	Low
Parent	Ilan	M	56	Omon’s son	Han	High school	Banner enterprise	Partial comprehension; simple speech	Multiethnic schooling	Moderate
Parent	Dijin	F	60	Omon’s daughter	Han	High school	Banner public institution (retired)	Comprehension; simple speech	Multiethnic schooling	Moderate
Grandchild	Nadan	M	34	Ilan’s son	Daur	University	Banner government	Minimal isolated words	University outside banner; returned for employment	High
Grandchild	Jejin	M	32	Dijin’s son	Unmarried	University	Banner public institution	Minimal isolated words	University outside banner; returned for employment	High

### 3.4 Photo materials: sources and selection principles

The photographic materials came from two sources. The first source consisted of photographs selected by the participants themselves. One week before the interview, each participant was invited to bring one or two photographs that, in their view, best represented their Oroqen identity. All five participants did so, and four voluntarily brought two photographs each. This open-ended prompt was intended to capture participants’ spontaneous identity narratives while minimizing the researcher’s prior assumptions.

The second source was a standardized photo set compiled by the researcher on the basis of a decade of fieldwork and existing scholarship on Oroqen culture. The set included 16 photographs covering key themes in Oroqen cultural life (see Supplementary material). These images came from the researcher’s own fieldwork photography, publicly available historical photographs from official Chinese media and the Inner Mongolia multimedia resource database for smaller ethnic groups, and official short video content released by the Oroqen Autonomous Banner government. All photographs were used for academic research under fair use principles and with standard attribution.

The 16 photographs were organized into three categories. Traditional and ecological life included six photographs: (a) the traditional Oroqen dwelling (*ʃɛɛran* *tʃʊʊ*); (b) hunters kneeling in worship of the mountain spirit; (c) an Oroqen woman on horseback carrying an infant in a traditional cradle; (d) a traditional wooden-handled hunting knife; (e) women processing animal hides; and (f) hunters returning from a hunt. Transition and settlement included four photographs: (g) children reading pictorial magazines in a classroom during the early settlement period; (h) a hunters’ village built under the 1953 sedentarization policy; (i) a teacher leading students in ethnic dance during the early settlement period; and (j) the 1996 hunting ban assembly. Reconstruction and modernity included six photographs: (k) a contemporary Oroqen language classroom; (l) a shamanic ritual performance; (m) a bear dance at the 60th anniversary of the banner’s founding; (n) an aerial photograph of a new hunters’ village; (o) a young Oroqen online content creator promoting their hometown; and (p) an Oroqen cultural outreach team being interviewed in another province. The three categories were balanced in number to avoid bias toward either traditional or contemporary representations.

The researcher recognized that the 16 standardized photographs offered a particular visual framing of Oroqen culture and could shape participants’ responses. Several steps were taken to reduce this effect. The three categories were represented equally. Participants were explicitly told that they could decline any photograph and were welcome to offer critical comments. In the analysis, particular attention was paid to photographs that participants did not select and to their critical responses. This helped identify identity resources that lay beyond the researcher’s initial assumptions. Each participant was asked to select the six photographs that resonated most strongly with them and then rank them by personal significance.

### 3.5 Interview procedure

One week before the formal interview, the researcher met with each participant individually to explain the purpose and procedures of the study, obtain verbal informed consent, and ask them to prepare their self-selected photographs. The content of the standardized photo set was not disclosed in advance.

The formal interviews were conducted in March 2026 in the participants’ homes. Each participant took part in one in-depth interview lasting 60 to 120 min. All interviews were audio recorded with the participants’ consent and later transcribed. The interviews were conducted primarily in Mandarin Chinese, and Oroqen words and phrases were preserved and annotated in the transcripts. Each interview proceeded in three phases.

In Phase 1, the participant discussed their self-selected photographs, including why they had chosen them, the context in which they were taken, and their personal meaning and associated emotions. In Phase 2, the researcher laid out all 16 standardized photographs and invited the participant to look through them, choose the six that resonated most strongly, and rank them by personal significance. Follow-up questions explored why certain photographs were chosen and why others were not. In Phase 3, the interview moved into a more in-depth narrative form. Following the participant’s ranking, the researcher explored the memories, emotions, and associations evoked by each photograph, and then broadened the discussion to language use, identity, intergenerational transmission, and hopes for the future.

Throughout the interviews, the researcher used open-ended follow-up questions and empathic responses to encourage participants to develop their narratives in their own way and at their own pace. When participants fell silent for an extended period or showed signs of emotional distress, the researcher waited and did not press for an immediate response. The interviews were conducted in a relaxed and informal atmosphere.

### 3.6 Photo selection records and data management

Table 2 presents each participant’s self-selected photographs, along with their selections from the standardized photo set and the rankings they assigned to them. All audio recordings were transcribed verbatim by the researcher. Oroqen language segments were transcribed using the International Phonetic Alphabet and accompanied by Chinese semantic glosses. Their accuracy was checked in consultation with older family members who were proficient in Oroqen.

**TABLE 2:** Self-selected photographs and standardized photo selection and ranking.

Pseudonym	Self-selected photos	Content of self-selected photos	Standardized photo selection and Ranking
Omon	2	Deceased husband in ethnic dress with hunting knife and horse (one at traditional Oroqen dwelling *ʃɛɛran* *tʃʊʊ*, one at post-sedentarization house)	e-a-c-j-m-g
Ilan	2	ID photo at age 5 with green military cap; family portrait at age 10 (including cousins)	j-a-d-g-e-f
Dijin	2	Recent photo in ethnic dress at the traditional Oroqen dwelling; ethnic dress photo taken in Tibet	d-b-e-f-g-i
Nadan	2	On horseback in ethnic dress in forest; WeChat profile photo in ethnic dress	d-f-a-b-m-k
Jejin	1	On horseback in ethnic dress during travel in Qinghai	j-i-o-p-g-m

The photographs and de-identified interview transcripts that support the findings of this study are available from the corresponding author upon reasonable request.

### 3.7 Data analysis

Data analysis followed Braun and Clarke (2006, 2019) six-phase reflexive thematic analysis, combined with elements of narrative structural analysis (Josselson and Hammack, 2021). The analysis began with familiarization, achieved through repeated reading of the interview transcripts and review of the elicited photographs alongside field notes. Initial codes were then generated inductively at the semantic level while remaining sensitive to the theoretical framework outlined in Section “2 Theoretical framework”; this open coding process yielded 379 initial codes closely grounded in the raw data. Each code was assigned a participant-specific prefix and sequential number, enabling each code to be traced back to its source participant and generation. Related codes were subsequently clustered into candidate themes through a combination of manual annotation, resulting in 29 descriptive categories. These candidate categories were then systematically reviewed against the coded extracts and the full dataset, moving from within-generation analysis to cross-generational comparison, in order to check internal coherence and distinctiveness. Themes were further refined, named, and defined through iterative reflexive memo-writing, with attention to how the author’s own positionality might shape interpretation; this process led to three core themes: from lived experience to memory resources; language boundaries, emotional burdens, and de-essentialized identity; and digital media and identity reconstruction in contexts of public display. Table 3 presents representative coding examples. Finally, the refined themes were integrated into the narrative reporting presented in the Findings section. Throughout, the process remained iterative rather than linear, with the researcher moving back and forth between phases as understanding of the data deepened. Preliminary findings were also shared with participants for comment and feedback, which strengthened the trustworthiness of the analysis.

**TABLE 3:** Representative coding examples.

Original quotation	Initial code	Descriptive grouping	Core theme
“*I was born in this kind of dwelling. Of course I lived in one.*”	Birth in a *ʃɛɛran tʃʊʊ*	Experiences and perceptions of the *ʃɛɛran tʃʊʊ*	From lived experience to memory resources
“*This is about the hunting ban. They stopped us from hunting. It did not feel good.*”	Feelings evoked by the hunting ban photograph	Sedentarization and the hunting ban as historical turning points	From lived experience to memory resources
“*Oroqen is the mother tongue of the Oroqen people. At the very least, one should be able to speak a little of it and not forget it.*”	Mother tongue as a basic requirement of identity	Intergenerational hopes for language transmission and the emotional burden it carries	Language boundaries, emotional burdens, and de-essentialized identity
“*That is certain. A hundred years from now, the Oroqen language will be gone. It will have become history.*”	Prediction of language extinction within a century	Structural barriers to language transmission	Language boundaries, emotional burdens, and de-essentialized identity
“*Let Oroqen culture go beyond Inner Mongolia, and even beyond China, to reach the wider world.*”	Online creators bringing Oroqen culture to a wider audience	Cultural communication and visibility in digital media	Digital media and identity reconstruction in contexts of public display

All interviews were conducted, transcribed, and coded by the sole author, who also carried out the fieldwork described in Section “3.2 Research setting, family background, and fieldwork foundation”. The final themes were determined by the author. To strengthen the trustworthiness of the findings, the preliminary thematic framework was reviewed by two colleagues familiar with Oroqen studies, who provided advisory feedback that the author considered in refining the themes; these colleagues did not conduct independent coding of the data.

### 3.8 Ethical considerations

This study received ethical approval from the researcher’s institutional review board. All participants provided written informed consent for the interviews, audio recording, the use of photographic materials, and the publication of findings. Given the small size of the Oroqen population and the close-knit nature of the community, even pseudonymized information may still make participants identifiable through details such as age, kinship relations, occupation, and life history. Accordingly, some background details have been withheld or blurred, while information essential to the analysis has been retained. To protect family privacy, participants’ self-selected photographs are presented as written descriptions rather than reproduced as images. The use of photographs involving deceased family members was approved with the family’s explicit consent.

This study is guided by the ethical view that identity constructions across generations are equally legitimate and that different generations live out their Oroqen identity in different ways. The findings will be shared with the participating family and the local community in a simplified Chinese version. Family members will also have an opportunity to suggest revisions before the final version is made public.

## 4 Findings

The findings are organized around three core themes, each showing how the three generations responded in different ways. Throughout the analysis, the focus is on generational tendencies rather than fixed generational types. The argument is not that every member of a generation fits a single identity pattern, but that a more prominent tendency can be seen within this family.

### 4.1 Theme 1: from lived experience to memory resources

The first theme examines how participants drew on traditional Oroqen ways of life in narrating who they were. Across the three generations, there was a clear shift from embodied living, to the keeping of memory, to narrative reconstruction.

#### 4.1.1 The grandparent generation: Oroqen identity as lifeworld

Omon, the only family member who had fully lived through the hunting and gathering life before sedentarization, chose two photographs of her late husband as her self-selected images. He had been a skilled Oroqen hunter. In one photograph, he stood in ethnic dress before a traditional Oroqen dwelling, with a hunting knife at his waist and a horse beside him. In the other, he wore everyday clothes and stood in front of a house built after sedentarization, still wearing the hunting knife.

This choice suggests that Omon understood her Oroqen identity in relational terms. She expressed it through her late husband rather than by speaking directly about herself. This should not be read as self-negation, but as a relational way of being Oroqen. Her sense of being Oroqen took shape through the life she shared with her husband. Placed side by side, the two photographs suggest that even after the shift in livelihood, embodied traditional practice remained central to her sense of self.

From the standardized set, Omon selected photographs e, a, c, j, m, and g, most of which centered on embodied practices and scenes from everyday life in the past. When discussing her top-ranked photograph, which showed women processing animal hides, she said:

*I remember everyone working on hides together. It was nice. That was during the cooperative period, around 1958, when the hunters’ village organized collective labor in exchange for work points. Those older women must have been in good spirits, chatting away in Oroqen.*

In this account, work, sociality, and joy are closely tied together. Looking at the photograph of the traditional Oroqen dwelling (*ʃɛɛran tʃʊʊ*), she said:

*I was born in this kind of dwelling. Of course I lived in one. This looks like a summer one. In winter they were not so tall. In summer they used birch bark. In other seasons they used animal hides.*

Her memory of this dwelling was not simply one of having seen it, but of having come from it. This is a vivid example of what Husserl called the *Lebenswelt*, or lifeworld: direct experience before it is turned into an object of reflection (Ni, 2019), marked by sensory detail and a strong sense of bodily belonging.

She ranked the photograph of the 1996 hunting ban fourth. Her response was ambivalent. She recognized why the policy was introduced, but she also spoke with a deep sense of loss.

*They stopped us completely. At that time, we hunted to feed ourselves.*

Here, hunting appeared not as an abstract cultural symbol, but as a basic means of survival. When speaking about contemporary shamanic performances, Omon drew a clear distinction: “*In the past, shamans healed the sick. Now these shamans have all become modernized.*” This suggests the perspective of a traditional insider on cultural authenticity. She could recognize these contemporary reconstructions, but did not fully accept them as the same thing.

Taken together, Omon’s sense of identity was firmly grounded in embodied practice and lived memory. For her, being Oroqen was a natural part of the world she had lived in, and it required neither explanation nor justification.

#### 4.1.2 The parent generation: Oroqen identity as transitional experience and memory guardianship

The two participants in the parent generation, Ilan and Dijin, were both born after sedentarization. Although they still had some contact with traditional Oroqen ways of life in childhood, they were mainly socialized in multiethnic settings shaped by Han dominance. For them, being Oroqen was neither a directly lived lifeworld nor a purely symbolic identity. Instead, it occupied an in-between position: no longer fully present, yet not entirely lost.

Ilan’s account of language use across home and school captures this transitional condition well. When asked whether he had spoken Chinese before starting elementary school, he replied:

*I only spoke Oroqen. After starting school, I didn’t speak Oroqen at school. I spoke it when I came home.*

This reflects a broader generational pattern. Mandarin belonged to school and the public sphere, while Oroqen remained in the home and the private sphere. This division also points to a larger problem in language transmission, as the home gradually comes under the pressure of the dominant language.

Ilan’s self-selected photographs, a childhood ID photo taken at age five and a family portrait taken at age ten, contained no visible Oroqen markers. Their meaning as Oroqen photographs depended entirely on the ethnic identity of the people in them. This visual pattern, inwardly Oroqen but outwardly ordinary, captures the transitional character of identity in this generation. When asked why he had chosen these photographs, Ilan gave a brief reply:

*Back then, at home, we all still spoke Oroqen.*

This response linked Oroqen identity to childhood, home, and language, evoking something like an inner homeland that was distant but still reachable.

Ilan ranked the hunting ban photograph first in the standardized set. His first response was brief:

*It’s about the hunting ban. They stopped us from hunting.*

When asked how he felt about it, he replied after a pause:

*[pause] Not a good feeling.*

The brevity of this response is itself meaningful. Behind the silence and the few words lies a deep sense of loss shared by many Oroqen men of his generation. The 1996 ban ended not only an economic activity, but also a practical bond between men of this generation and their fathers. His third-ranked selection, the hunting knife photograph, revealed a similar sense of being caught in transition. When asked whether he still used such a knife, he replied:

*Not much now. Sometimes. But very rarely.*

When asked how he would feel if he saw it in a museum, he said:

*Excited!*

But when asked what else he felt, he fell silent. This silence after the emotional outburst reflects what Pugh (2013) described as visceral experience: affect felt at the bodily level before it becomes fully available in words. Men of this generation could no longer use the hunting knife as an everyday tool, as the grandparent generation did, but had not yet turned it into a cultural symbol in public narratives, as the grandchild generation would. They seemed to inhabit an affective space between tool and symbol, one that had not yet found full expression in language.

Dijin’s transitional experience took a rather different form. Now retired and actively involved in cultural display activities, she selected a photograph of herself in ethnic dress during a tourist reception, with a traditional Oroqen dwelling in the background. She explained:

*Last winter, I was receiving tourists at the Uliren site. I was wearing a hat with Oroqen features and ethnic clothing. The tourists were all from the south, from places like Guangdong and Guangxi. It was to promote Oroqen culture.*

Dijin’s account points to an identity shaped not only by inner belonging, but also by public display. Importantly, this orientation toward public display did not belong only to the grandchild generation. It had already emerged in a woman of the parent generation, which complicates any simple account of a sharp generational break.

Dijin’s photograph selections (d, b, e, f, g, and i) also revealed a gendered dimension in the making of Oroqen identity. While Ilan’s core reference points centered on masculinized practices such as hunting and the hunting knife, Dijin’s selections highlighted women’s traditional labor and ritual practices, including mountain spirit worship, hide processing, dance, and child-rearing. Within this family, Oroqen identity was not a single, uniform experience, but was differentiated by gender into distinct domains of practice and memory. Compared with Ilan, Dijin took a more proactive stance toward the younger generation:

*I think he [her son Jejin] should at least be able to understand some simple Oroqen words when I say them at home. At the very least, he should learn to say a few simple Oroqen phrases. Otherwise, how can he say he is Oroqen? At the very least, he should be able to understand it.*

The repeated phrase “*at the very least*” suggests a strategy of lowering expectations while still holding on to a basic standard. It reflects the parent generation’s pragmatism and protective instinct in passing on culture.

The parent generation’s sense of identity centered on lived memory and historical experience, while in some cases, as with Dijin, it also extended toward symbolic display. Their way of being Oroqen was grounded in family memory and in witnessing historical change. They were keenly aware of cultural loss, yet also felt a strong moral responsibility to preserve memory. In this sense, their narratives resembled what Elbaz and Wiseman (2026) call the “retelling the past” pattern.

#### 4.1.3 The grandchild generation: Oroqen identity as a narrative object

Both participants in the grandchild generation were born after the 1990s and had never directly experienced the hunting life. Their sense of being Oroqen was shaped mainly through family narratives, school education, official discourse, and media representations. Yet it would be misleading to describe them simply as “symbolic” or “performative.” Their identity work also involved genuine emotional attachment, critical reflection, and a sense of cultural responsibility.

Nadan’s self-selected photograph showed him in ethnic dress on horseback in a forest. The combination of ethnic dress, horse, and forest created an image of the “idealized Oroqen.” This was not part of Nadan’s everyday life, but something deliberately assembled for special occasions such as wedding rituals and ethnic cultural events. When asked whether there was a moment when he felt especially Oroqen, Nadan replied:

*The wedding, for sure. When we got married, we rode horses up the mountain. That’s something I’ll never forget. We were both in ethnic dress. Nowadays, who rides a horse at their wedding? Everyone takes cars to the hotel banquet hall. We rode up on horseback.*

By bringing horses and ethnic dress into his wedding, Nadan turned a major life event into a powerful expression of Oroqen identity. This shows a characteristic strategy in the grandchild generation: using traditional symbols at key life moments to create especially intense experiences of identity. The emotional investment here was clearly genuine. Nadan’s statement that he would never forget the horseback wedding scene points to a deeply felt attachment.

Nadan also described an experience that revealed the reactive side of identity formation in the grandchild generation:

*Right after I got my job in the banner, people from my work unit went to my university for a background check. Some people there didn’t know what the Oroqen Banner was. If this happens again, I will definitely use our ethnic culture and history to tell them about it. Even though we no longer speak Oroqen, we still need to have ethnic pride.*

When people outside failed to recognize the Oroqen Banner, Nadan felt a sharp gap in recognition, which he turned into a more active commitment to explaining and representing his culture. This experience further strengthened his engagement with ethnic dress, traditional rituals, and cultural history. His photograph ranking (d, f, a, b, m, and k) reflected this orientation, with the top four places all given to photographs of traditional practices. The loss of language was partly offset through other cultural resources, including clothing, ritual, and history. This formed a key logic of identity construction that was no longer based on language alone.

Jejin’s self-selected photograph was taken beneath the snowy mountains of Qinghai Province and showed him in ethnic dress on horseback. Compared with Nadan’s selection, Jejin’s photograph was transregional. He carried Oroqen symbols beyond their original geographic setting, showing how identity in the grandchild generation was no longer tied to one settlement, but could be expressed across different places.

Jejin’s photograph ranking was j, i, o, p, g, and m. Like his uncle Ilan, he placed the hunting ban photograph first, but interpreted it in a very different way:

*We could adapt to settled life down in the valleys. We said goodbye to that nomadic, unsettled hunting life. Step by step, through rapid development, we arrived where we are today.*

Where Ilan spoke of “*not a good feeling*,” Jejin spoke of “*step by step*” development. This difference cannot be explained simply by age or personality. It is rooted in the very different historical worlds each generation has lived through. Jejin had never experienced the hunting life, and for him the ban meant not loss, but progress.

Taken together, the grandchild generation’s sense of identity centered on symbolic display and digital media. Their identity was expressed through visual symbols and public narratives, and it was marked by a strong sense of speaking for the group, along with an ability to reflect critically on culture and history.

#### 4.1.4 Summary of theme 1

Across the three generations, there was a clear shift from embodied life, to memory as a cultural resource, to narrative reconstruction, although this shift was neither linear nor absolute. Ilan still retained traces of embodied experience, as shown in his silence after his emotional response to the hunting knife. Dijin had already moved toward a more publicly displayed form of identity. Nadan’s vivid memory of the horseback wedding revealed an emotional authenticity that went beyond mere symbolization. Omon’s critical assessment of contemporary shamanic performances also showed a clear awareness of cultural reconstruction. For these reasons, the pattern identified here is better understood as a generational tendency rather than a fixed generational type.

### 4.2 Theme 2: language boundaries, emotional burdens, and de-essentialized identity

The second core theme concerns the complex relationship between language competence and ethnic identity. The Oroqen language is severely endangered (Moseley, 2010), and this is reflected in the clear generational gradient in language competence. Yet this gradient is not only a matter of linguistic ability. It is also a field of emotion, value judgment, and cultural concern.

#### 4.2.1 The grandparent generation: language guardianship and essentialist judgments

Omon, the family’s only fluent speaker of Oroqen, stated:

*If you can’t even speak Oroqen, what kind of Oroqen are you? You’ve thrown away your own language, can you still be called Oroqen?*

This reflects a strongly essentialist view of the link between language and identity (Debenport and Webster, 2019). However, this should not be read simply as prejudice. It is better understood as a fluent speaker’s response to the pain of language loss, and as a deep anxiety expressed in stark terms. At the same time, Omon also showed a more accommodating side:

*If they can speak a few phrases, then a few phrases, otherwise it’ll be nothing at all.*

This shift from strictness to accommodation reveals a tension in Omon’s view of language. She insisted on a deep connection between language and identity, yet also recognized the need for compromise under current conditions.

#### 4.2.2 The parent generation: linguistic separation across spaces and compromise

Both Ilan and Dijin could understand Oroqen and produce simple utterances, but could not sustain complex conversations. Their linguistic experience followed a clear spatial division: Mandarin for school, work, and public life, and Oroqen for home, elders, and private life. This arrangement was workable in childhood, but it gradually weakened as Mandarin entered the home through Han spouses and children’s schooling. As a result, Oroqen competence declined sharply in the next generation.

The parent generation’s expectations around language show a strategy of compromise, best captured in Dijin’s repeated phrase “*at the very least*.” She did not demand fluency, but she did insist that basic comprehension should remain the minimum standard. This position stands between the grandparent generation’s more essentialist judgment and the grandchild generation’s looser understanding of identity. It reflects a generational ethic in which the older generation remains responsible for passing on at least a minimal linguistic bond, while the younger generation is expected to receive it, even imperfectly. This echoes Hinton’s (2001) idea of “fragmentary transmission.”

#### 4.2.3 The grandchild generation: language limitations and alternative identity construction

Both participants in the grandchild generation understood only a limited number of Oroqen words. They acknowledged that their generation could no longer speak Oroqen, yet maintained that this did not diminish their ethnic identity, which could still be expressed through other means such as ethnic dress and cultural performance.

When asked how he would feel if no one spoke Oroqen in the future, Jejin responded in striking contrast to Omon:

*No particular feeling, because the ethnic group still exists. It can be displayed through folk songs, ethnic dress, and technological means like AI and 3D imaging. The flood of history rolls forward; no one can stop it.*

Through a language of technology and historical inevitability, Jejin recast what Omon saw as ethnic disappearance as the continuation of the group in new forms. He also spoke about language learning:

*If you want to learn Oroqen, personal demonstration and immersive teaching would probably work better. It has to be integrated with our lives.*

This suggests an understanding that, as an unwritten language, Oroqen can only be passed on effectively through embodied practice. In this sense, Jejin’s view echoed the grandparent generation’s embodied approach to language, although he expressed it in a much more explicit and reflective way.

#### 4.2.4 Summary of theme 2

All three generations recognized the loss of language, but their emotional responses and ways of dealing with it differed considerably. The grandparent generation expressed this loss through essentialist language, the parent generation responded with compromise, and the grandchild generation reframed it through technology and a sense of historical inevitability. What they said about the Oroqen language reflected not only their different levels of competence, but also deeply held views about language, shaped by emotional investment, ethical judgment, and cultural imagination. These findings challenge the essentialist assumption that declining language competence necessarily leads to weakened identity. Language loss does erode cultural memory and ways of understanding the world (Whorf, 1956; Lucy, 1992), but identity does not disappear with it. Instead, it finds other anchors.

### 4.3 Theme 3: digital media and identity reconstruction in contexts of public display

The third core theme concerns digital media, public display, and the reshaping of identity through media. This theme emerged most clearly in the accounts of the two participants from the grandchild generation, but it also brought out the different attitudes of the parent and grandparent generations.

#### 4.3.1 The grandchild generation’s digital identity practices

Digital media and public communication appeared repeatedly in the interviews with the grandchild generation. Both participants took a nuanced view of Oroqen internet celebrities. They valued the online sharing of Oroqen culture, but also criticized content that they saw as driven only by online traffic and lacking cultural depth.

*Let everyone know about our Oroqen culture, get our culture out there. If I find something said incorrectly, I’ll send them a private message to tell them. (Jejin)*

*Some videos are quite well made. But some are just chasing traffic and don’t match our culture. Ethnic cultural communication must be done by people who understand and love the ethnic group. (Nadan)*

This stance of critical engagement shows the grandchild generation’s capacity for reflective judgment in media-based identity work. They were neither passive media consumers nor simple cultural performers. Instead, they acted as thoughtful cultural representatives who evaluated media content even as they participated in it.

Both participants also said that they sometimes reposted Oroqen-related content on platforms such as WeChat Moments and short-video accounts, and occasionally made their own videos. They did not see these posts mainly as staged performances, but as records of everyday Oroqen life in the present. This layered form of media practice combined the roles of cultural communication and everyday documentation. It also challenges the assumption that engagement with media is always performative (Schechner, 2013).

#### 4.3.2 The grandparent and parent generations: spectators and participants

Omon kept a certain distance from contemporary media representations of Oroqen culture. She appreciated them, but did not fully identify with them. Ilan responded in a similar way to young Oroqen internet celebrities. He recognized their value in spreading Oroqen culture, but also felt some unease about their distance from tradition.

*It’s okay. The communication is a bit too modernized. (Ilan)*

Dijin, by contrast, expressed strong support:

*These kids are doing a great job, promoting our own ethnic culture with real boldness. Otherwise, who would even know about the Oroqen? (Dijin)*

Drawing on her own experience of presenting culture to others, Dijin was keenly aware of the visibility problems faced by a small ethnic group that most outsiders knew very little about. From this perspective, she welcomed almost any form of public communication, even when it relied more on visible symbols than on the Oroqen language itself. Her stance suggests that a public, display-oriented approach cannot be explained by generation alone, but is also closely tied to a person’s social role and lived experience.

In sum, the reshaping of Oroqen identity in digital media shows how culture and personhood continue to shape each other in the new media environment. Media gave people new ways to express culture, while their own media practices also reproduced and transformed cultural forms. The grandchild generation’s digital media practices should not be reduced to symbolic display alone. They reflect a more complex form of identity work that moves across several tensions, including emotional authenticity, speaking for the group, and personal expression.

## 5 Discussion

Using a single-family case study and photo-elicited nostalgic narratives, this study examined how identity anchors were passed across generations in an Oroqen family and considered the implications for cultural psychology. The following discussion is organized around three key findings.

### 5.1 Compressed cultural change and the generational transfer of identity anchors: extending the mutual constitution principle

The central finding is that, across only three generations and roughly eight decades of rapid cultural change, Oroqen identity anchors shifted from embodied experience to memory guardianship and then to narrative reconstruction. This finding extends the mutual constitution principle (Markus and Kitayama, 1991, 2010; Shweder, 1991). Mutual constitution operates not only between culture and individuals, but also across generations within the same cultural world, and it does so in different ways.

For the grandparent generation, identity anchors were rooted in an embodied lifeworld (Ni, 2019). For them, being Oroqen was not something to reflect on, but something lived as an ordinary whole. This resonates with Biddle and Swee’s (2012) finding that among Indigenous Australians, embodied ties to ancestral land and traditional subsistence practices are linked to subjective well-being. It also aligns with Pugh’s (2013) account of “visceral experience.” For Omon, Oroqen identity was a lived reality that could be narrated, not a trait that needed to be displayed or proven.

The parent generation occupied a more liminal position. From the perspective of self-continuity (Becker et al., 2018; Sedikides et al., 2015), they faced the greatest challenge in maintaining a coherent sense of self across time. The grandparent generation sustained continuity through rich embodied memory, and the grandchild generation rebuilt it through symbolic reconstruction. The parent generation, however, experienced the sharpest rupture between past and present. They witnessed the end of traditional life, yet had neither the immersive memories of the grandparent generation nor the alternative symbolic resources available to the grandchild generation.

Here, Sedikides et al.’s (2015) idea of nostalgia as a resource for restoring self-continuity takes on a clear generational dimension. For the parent generation, nostalgia was not simply an emotion. Through memory guardianship, it became a way to repair the continuity of self disrupted by institutional rupture. Elbaz and Wiseman (2026) described this process as “narrative reasoning of retelling the past,” while Camia and Zafar (2021) showed the protective value of autobiographical meaning-making among refugee populations. In this study, the parent generation similarly used memory guardianship as a psychological resource in the face of cultural rupture.

In the grandchild generation, identity anchors shifted further toward narrative reconstruction and symbolic display, but their identity work should not be reduced to performance or symbolism alone. Nadan’s emotional investment in his wedding memories, Jejin’s expressions of ethnic pride, and their critical engagement with online celebrity culture all point to a more complex negotiation across several tensions. This challenges Schechner (2013) tendency to equate media performance with performative practice, and it also speaks to Van Dijck’s (2008) account of digital photography’s shift from a tool of memory to a tool of identity construction. In the grandchild generation’s practices, photographs served both as carriers of memory and as expressions of identity, often at the same time and within the same digital contexts.

From the perspective of intergenerational synergy, this family developed a distinctive identity ecology. The grandparent generation provided experiential authority, the parent generation took on the moral task of guarding memory, and the grandchild generation carried culture into public communication and creative adaptation. This ecology was not a neat division of labor, but a dynamic balance marked by internal tensions. Together, these roles formed the family’s complex landscape of cultural negotiation.

From a cultural psychological perspective, this identity ecology adds a generational dimension to the mutual constitution principle. Culture is reproduced not only through individual practice, but also across generations in differentiated, complementary, and mutually supportive ways. This finding resonates with Schwartz et al.’s (2020) call for closer dialogue between cultural psychology and developmental science, and it also fits with Zilber et al.’s (2008) embedded narrative theory.

### 5.2 The emotional weight of language boundaries: from essentialist to de-essentialized identity construction

The second core finding concerns the complex relationship between language ability and ethnic identity. When asked whether a person can still be Oroqen without speaking the language, the three generations gave sharply different but equally thoughtful answers. Together, their responses formed a cross-generational dialogue about language and identity.

Omon’s essentialist position reflects what Debenport and Webster (2019) called “strategic essentialism.” This was not simply a philosophical claim about language, but a form of discursive resistance in the face of language loss. The shift in her account from firmness to accommodation reveals the inner tension within this form of strategic essentialism.

The parent generation occupied a middle position between the grandparent generation’s essentialism and the grandchild generation’s more de-essentialized view, echoing Hinton’s (2001) idea of “fragmentary transmission.” Yet the data also suggest that this partial transmission faced deep structural obstacles. Ilan’s description of a split language environment shows how the private space for using Oroqen narrowed sharply under the combined pressures of intermarriage and Mandarin-medium schooling. This aligns with Bromham et al.’s (2020) study of Gurindji, which found that speaking ability declines much faster than receptive ability, and that peer groups are the strongest factor shaping language variation.

Grenoble and Whaley’s (1999) comparison of Chinese and Soviet policies toward Tungusic languages revealed a striking paradox. In China, relatively accommodative ethnic policies encouraged interethnic marriage and wider social integration, but also unintentionally accelerated language assimilation. By contrast, the Soviet Union’s more coercive policies toward the Evenki fostered a more oppositional ethnic identity and in this way partly slowed language loss. The changing ethnic backgrounds of spouses across the three generations in this family, from Oroqen to Han to Daur, offer a micro-level illustration of this broader paradox. In this sense, the very success of accommodative policies in education, employment, and economic life helped create the conditions for interethnic marriage and social integration, which then became structural barriers to language transmission.

The grandchild generation’s more de-essentialized approach to identity deserves particular attention. Nadan’s and Jejin’s statements should not be read as signs of indifference to language. Rather, they reflect alternative ways of building identity under conditions of limited language ability. Neville et al.’s (2014) framework of five dimensions of belonging, and the compensatory relations among them, offers a useful cross-cultural lens here. It helps explain how the Oroqen grandchild generation builds belonging through other anchors, such as ethnic dress, ritual performance, and digital communication, when shared language is structurally out of reach.

Whether this more de-essentialized form of identity can sustain a strong sense of ethnic belonging over the long term remains an open empirical question. In the short term, the adaptive value of Jejin’s technological optimism and historical fatalism should not be underestimated. Over the longer term, however, if the ecological knowledge, kinship terms, and ways of understanding space that are deeply embedded in a language (Whorf, 1956; Lucy, 1992) disappear along with the language itself, the lived richness of ethnic identity may be irreversibly thinned. As Janhunen (2005) observed, language loss can involve the breakdown of a broader ethnic worldview and cognitive system.

### 5.3 Digital media and identity reconstruction: the visibility crisis and a three-generation identity ecology

The third core finding concerns digital media as an emerging context for identity construction. With a population of fewer than ten thousand, the Oroqen face a serious visibility problem, as most outsiders know very little about them. As Dijin put it, “*Otherwise, who would even know about the Oroqen?*”

Van Dijck (2008) noted that digital photography has shifted from a tool of memory to a means of identity construction and communication. The media practices of the grandchild generation provide support for this claim in an endangered cultural context. However, the present study suggests a more complex process than the one-way shift emphasized by Van Dijck. In endangered cultural contexts, digital media practices can serve two closely linked functions at once. One is inward-looking: documenting the lives of the grandparent generation and preserving cultural images. The other is outward-looking: communicating identity and making the Oroqen visible to the wider world. These two functions are not necessarily in tension. On the same media platforms, they often coexist and support each other.

The grandchild generation’s critical stance toward internet celebrity culture points to a more complex form of mediatized identity. They are not passive media consumers. Instead, they act as cultural gatekeepers who evaluate and, when necessary, correct media content about their ethnic group. This reflective form of media participation challenges the stereotype that digital natives engage with media only through shallow consumption or symbolic display. It is also important to note that the grandchild generation has, in effect, taken over a gatekeeping role once held by elders and other cultural authorities. This role has shifted across generations in part because many elders are themselves unfamiliar with digital media.

Omon’s distance as a spectator from contemporary mediatized Oroqen culture stands in clear contrast to the grandchild generation’s active communicative engagement. This contrast reflects generational differences in what counts as authentic Oroqen culture. For Omon, cultural authenticity is grounded in embodied experience and historical memory. For the grandchild generation, cultural meaning depends more on present communicative reach and emotional resonance. This generational shift in the criteria of cultural judgment offers a distinctive expression of the mutual constitution principle in the age of mediatization. Culture is not only reproduced through practice; the authority to define culture is also redistributed across generations.

Viewed through the lens of a three-generation identity ecology, the study reveals a pattern in which experiential depth declines as communicative breadth expands. The grandparent generation holds a depth of cultural experience that later generations cannot reproduce, yet their cultural expression remains largely confined to the family and local community. The grandchild generation’s cultural experience is thinner because of language loss and distance from traditional ways of life, but through digital media they have extended the reach of Oroqen cultural communication in unprecedented ways. The parent generation occupies an intermediate position in this pattern. The identity work of the three generations does not form separate or competing paths. Instead, it reflects complementary positions within a shared identity ecology. This finding offers evidence from an intense case of cultural change for understanding the generational and media dimensions of the mutual constitution principle.

Finally, the study also has several methodological implications. Photo-elicitation interviewing, or PEI (Harper, 2002; Clark-Ibáñez, 2004), proved especially valuable for capturing generational differences in identity construction within an endangered cultural context. As visual anchors, photographs effectively drew out emotional responses and forms of narrative reasoning grounded in the distinct life experiences of each generation. Different interpretations of the same photograph produced some of the most vivid material for understanding generational differences in identity. This methodological contribution also supports Pink (2013) view of the value of visual ethnographic methods in cultural research. At the same time, this study suggests that when PEI is used in cross-generational research, careful attention is needed to how the researcher’s choice of images shapes participant narratives. This methodological issue has received too little attention in the existing literature and deserves further examination.

### 5.4 Limitations and future directions

This study has several limitations that should be acknowledged. As a single-family case study, this research captures only one possible pattern of intergenerational transfer in identity anchors. It does not aim to support broad generalizations about the Oroqen as a whole or about Chinese ethnic minorities more generally. The value of PEI also depends on how individual participants respond to visual materials. The same set of photographs may draw out very different identity narratives in different families. Although reflexive analysis and member checking were carried out, the coding was not independently checked by a second researcher. The researcher’s long-term relationship with the participating family enriched the depth of the interviews, but it may also have shaped what participants chose to present, given their familiarity with the researcher. Finally, this study captured differences in identity anchors at one particular historical moment and did not follow how these dynamics changed over time.

Future research could build on these findings in several ways. Comparative work across multiple families would strengthen the transferability of the findings. Longitudinal designs could trace how identity anchors shift over the life course. Quantitative approaches could also be used to test more systematically the proposed pattern of diminishing depth and expanding breadth across generations. Another valuable direction would be to further develop methodological standards for PEI in cross-generational research, especially strategies for handling the framing effects of researcher-selected visual materials.

## 6 Conclusion

In an ethnic group that moved from forest hunting to digital society within just three generations, identity construction reveals a generational range rarely documented in cultural psychology. Omon experienced being Oroqen as a lifeworld that did not need to be explicitly stated. Ilan, caught between the excitement and the silence evoked by a hunting knife, held on to a childhood world that had already become distant but was not entirely lost. Nadan and Jejin, through a horseback wedding ritual and an ethnic-dress photograph taken beneath snowy mountains, have created forms of Oroqen identity expression shaped by the digital age. When looking at the same photograph of the hunting ban assembly, one generation felt “*not a good feeling*,” while another saw “*historical leapfrog development*.” This divergence does not reflect simple opposition. Rather, it shows how the same cultural resource takes on different meanings when viewed through the distinct life experiences of different generations. The mutual constitution principle thus takes on a generational dimension. Culture does not shape individuals as a fixed whole; instead, it is distributed, negotiated, and reworked differently across generations.

This study also reveals an important paradox. The very institutional arrangements intended to improve Oroqen well-being, including educational support, economic accommodation, and social integration, have also become structural barriers to intergenerational language transmission. Within this paradox, cultural transmission is no longer simply a matter of whether people want to preserve culture, but of how preservation is actually possible. When Omon asks, “*You’ve thrown away your own language, can you still be called Oroqen?*” and Jejin responds, “*The flood of history rolls forward; no one can stop it*,” they express not a simple difference between right and wrong, but different strategies for cultural continuity: essentialist persistence, pragmatic guardianship, and de-essentialized creation. Together, these strategies show the multiple pathways through which an endangered cultural community can sustain continuity under conditions of rapid change.

In this study, PEI served not only as a method of data collection, but also as a medium for intergenerational dialogue. The sixteen standardized photographs served as visual anchors for cultural symbols and brought three generations into a shared frame of reference. They made it possible to elicit, compare, and understand each generation’s life experience through the same visual prompts. This approach highlights the particular value of visual methods in research on endangered cultures. In a community undergoing language loss, photographs provide an emotional and mnemonic channel that can reach across linguistic barriers. Taken together, the photo narratives of the three generations show that identity construction is not about preserving a pure cultural form. It is about each generation answering the question of who they are in its own way, under its own historical conditions and within its own lifeworld. Each of these answers, whatever form it takes, is meaningful and deserves to be heard.

For cultural psychology, the Oroqen case invites a reconsideration of the boundary conditions of the mutual constitution principle. In a world of rapid cultural change, individuals and generational communities are not passive recipients when they confront the loss and reconstruction of cultural resources. They are active makers of meaning. The shifting of identity anchors does not necessarily signal the decline of identity. It may instead reflect its adaptive reconstruction in the face of historical rupture. This may be the deeper implication of the mutual constitution principle. The co-construction of culture and psyche occurs not only through the stable transmission of culture, but also, and often most vividly, in moments of cultural rupture and reconstruction.

## Data Availability

The original contributions presented in the study are included in the article, further inquiries can be directed to the corresponding author.
