# Supplementary material for: Generational compression and the shifting anchors of identity: a photo-elicitation nostalgia narrative study of identity construction across three generations in an Oroqen family
Source: Front Psychol. 2026 Sep 25;17:1915280. doi: 10.3389/fpsyg.2026.1915280 (PMC13649516; doi:10.3389/fpsyg.2026.1915280)
Supplement: Supplementary file 1 [file Table_1.DOCX]

**The 16 photographs were organized into three categories.**

**Traditional and ecological life included six photographs:**

(a) the traditional Oroqen dwelling (ʃɛɛran tʃʊʊ);

Source: Xinhuanet

URL: https://www.xinhuanet.com/photo/2021-08/11/c_1127752649_2.htm

(b) hunters kneeling in worship of the mountain spirit;

Source: Cultural Sharing Project of China- Inner Mongolia Cultural Information Network

URL: https://www.nmgcnt.com/tszy/ssmz/elcz/dweztp/ewkzzjxy/201510/t20151001_47824.htm

(c) an Oroqen woman on horseback carrying an infant in a traditional cradle;

Source: Cultural Sharing Project of China- Inner Mongolia Cultural Information Network

URL: https://www.nmgcnt.com/tszy/ssmz/elcz/dweztp/ewkzhsjq/201507/t20150723_46539.htm

(d) a traditional wooden-handled hunting knife;

Source: Cultural Sharing Project of China- Inner Mongolia Cultural Information Network

URL:

https://www.nmgcnt.com/tszy/ssmz/elcz/dweztp/ewkzjs/201509/t20150930_47802.htm

(e) women processing animal hides;

Source: Cultural Sharing Project of China- Inner Mongolia Cultural Information Network

URL: https://www.nmgcnt.com/tszy/ssmz/elcz/dweztp/ewkzhsjq/201507/t20150723_46531.htm

(f) hunters returning from a hunt.

Source: China Cultural Sharing Project · Multimedia Resource Database for the Four Small Ethnic Groups in Inner Mongolia (Offline Reading Terminals)

**Transition and settlement included four photographs:**

(g) children reading pictorial magazines in a classroom during the early settlement period;

Source: Xinhuanet Inner Mongolia Channel

URL:

http://www.nmg.xinhuanet.com/20241017/0c62861562264b33b12bbf59cbcc38e8/c.html

(h) a hunters’ village built under the 1953 sedentarization policy;

Source: Cultural Sharing Project of China- Inner Mongolia Cultural Information Network

URL: https://www.nmgcnt.com/tszy/ssmz/elcz/dweztp/ewkzfzxz11_1/201510/t20151005_48195.htm

(i) a teacher leading students in ethnic dance during the early settlement period;

Source: China Cultural Sharing Project · Multimedia Resource Database for the Four Small Ethnic Groups in Inner Mongolia (Offline Reading Terminals)

(j) the 1996 hunting ban assembly.

Source: Cultural Sharing Project of China- Inner Mongolia Cultural Information Network

URL: https://www.nmgcnt.com/tszy/ssmz/elcz/dweztp/ewkzfzxz11_1/201510/t20151005_48203.htm

**Reconstruction and modernity included six photographs:**

(k) a contemporary Oroqen language classroom;

Photograph taken by the researcher, August 2017

(l) a shamanic ritual performance;

Photograph taken by the researcher, August 2017

(m) a bear dance at the 60th anniversary of the banner’s founding;

Source: Cultural Sharing Project of China- Inner Mongolia Cultural Information Network

URL: https://www.nmgcnt.com/tszy/ssmz/elcz/dweztp/ewkzfzxz11_7419/201507/t20150725_46622.htm

(n) an aerial photograph of a new hunters’ village;

Source: Xinhuanet

URL: https://www.news.cn/politics/2021-10/28/c_1128006211.htm

(o) a young Oroqen online content creator promoting their hometown;

Source: Screenshot from the official Wechat video account of Oroqen Autonomous Banner People's Government, search date: February 10, 2026

(p) an Oroqen cultural outreach team being interviewed in another province.

Source: Screenshot from the official Wechat video account of Oroqen Autonomous Banner People's Government, search date: February 10, 2026
